# DuDeM: A Dual-Network Model for Early Gastric Cancer Detection Based on Capsule Endoscopy

**DOI:** 10.3390/bioengineering13030356

**Published:** 2026-03-18

**Authors:** Tianyi Feng, Qian He, Tianqi Chen, Weibing Wang

**Affiliations:** 1 Shanghai Institute of Infectious Disease and Biosecurity, Fudan University, Shanghai 200032, China; fengtianyi@caict.ac.cn; 2 China Academy of Information and Communications Technology, Beijing 100191, China; heqian1@caict.ac.cn (Q.H.); chentianqi@caict.ac.cn (T.C.); 3 Department of Epidemiology, School of Public Health, Fudan University, Shanghai 200032, China

**Keywords:** capsule endoscopy, early diagnosis, gastrointestinal endoscopy, deep learning

## Abstract

Early detection is critical for improving outcomes in gastric cancer, yet lesion recognition in capsule endoscopy is challenged by interference from different gastric anatomical sites, patient posture changes, and gastric peristalsis. This study aims to prompt a robust deep learning model to address these challenges. A dual-network model, named DuDeM (DualNet Detection Model), was developed by integrating a ResNet50-based convolutional branch with a CapsuleNet branch incorporating dynamic routing. The convolutional branch extracts local lesion features that are transmitted to primary capsules, while dynamic routing enables adaptive matching between capsule layers to establish local–global feature associations. An attention-weighted strategy is applied for feature fusion. The model was trained using capsule endoscopy images from nine hospitals in China and public datasets, and its performance was compared with eight representative models, with ablation analyses validating key components. Results showed that DuDeM achieved an area under the curve (AUC) of 0.981 and an F1-score of 0.979, with sensitivity, specificity, and precision all exceeding 97%, and performance degradation limited to within 3% under mild image perturbations. These findings suggest that DuDeM enables reliable early gastric cancer (EGC) recognition and may support large-scale capsule endoscopy screening in clinical practice.

## 1. Introduction

Gastric cancer remains a major global health burden, with a particularly high burden in East Asia, accounting for approximately 53.8% of global incident cases and 48.2% of deaths [1,2]. Given that early-stage gastric cancer is often asymptomatic, most patients are diagnosed at advanced stages. Research suggests that the five-year survival rate remains below 30% even after surgical treatment, while timely detection and intervention for early gastric cancer (EGC) can increase the five-year survival rate to over 90% [3]. Thus early screening and diagnosis of gastric cancer have become a consensus [4] and a cornerstone strategy for improving patient prognosis.

Endoscopy is one of the gold standards for early screening, and magnetically controlled capsule endoscopy (MCCE), due to its noninvasive nature, high patient acceptability, and scalability, has emerged as a promising tool for population-based screening [5,6,7,8]. However, capsule endoscopy in gastric cancer screening requires a high volume of images per patient [9], placing a substantial burden on manual interpretation. Prolonged reading time and reader fatigue significantly increase the risk of missed lesions, highlighting the need for automated assistance. In recent years, artificial intelligence (AI), particularly deep learning-based image analysis, has made substantial progress in gastrointestinal endoscopy [10].

Despite these advances, the application of AI-assisted capsule endoscopy for EGC screening remains insufficiently explored. Xu Zhang et al. reported that progress in deep learning-based gastric endoscopy substantially lags behind that in colorectal endoscopy [11], largely due to the greater anatomical complexity of the stomach and the pronounced heterogeneity of gastric images. A study by Xiaotong Wang et al., reviewing literature published between 2006 and 2022, further demonstrated that existing AI-assisted capsule endoscopy studies predominantly focused on gastric polyps, erosions, and ulcers while being scarce in gastrointestinal diseases screening [12]. Compared with the relatively tubular and structurally consistent colon, the stomach exhibits a wider lumen, complex mucosal folds, variable distension states, and dynamic peristalsis [13]. These characteristics lead to substantial variability in lesion appearance, viewing angles, illumination, and background context in capsule endoscopy images, posing distinct challenges for automated analysis. Most existing approaches rely on convolutional neural networks (CNNs) and their variants (ResNet50, YOLOv5, Faster R-CNN, etc.), or detection-oriented frameworks, which are effective in capturing local texture features or performing lesion localization [14,15,16]. However, such models have intrinsic limitations in modeling object pose, spatial configuration, and long-range contextual dependencies, resulting in reduced sensitivity and limited robustness when detecting small, low-contrast, and morphologically variable early gastric lesions.

Recent advances in capsule networks, which explicitly model part-whole spatial relationships through routing mechanisms [17,18], and CNN–Transformer hybrid architectures, which integrate local texture representation with global contextual modeling via attention [19], offer promising directions to address these limitations. Yet, evidence remains insufficient on how to integrate local texture and global pose-aware features in a unified framework while maintaining performance, robustness, and computational efficiency in capsule endoscopy screening.

To address these challenges, this study proposes a dual-network detection model, named DuDeM (DualNet Detection Model), specifically designed for EGC recognition in capsule endoscopy images. The proposed model integrates the complementary strengths of CNNs and CapsuleNets by combining a ResNet50-based convolutional branch for local feature extraction with a capsule-based branch for spatial and pose-aware representation learning. An attention-weighted fusion strategy is further employed to associate local texture features with global spatial information. The model is trained and evaluated using a multi-center capsule endoscopy dataset collected from nine tertiary hospitals in mainland China, reflecting real-world screening conditions. Performance is systematically compared with representative deep learning models, and ablation and robustness analyses are conducted to assess model effectiveness and clinical applicability.

## 2. Materials and Methods

### 2.1. Models

The methodology of this study focused on the construction of the DuDeM model, which uses ResNet50 model as the baseline and incorporates the strengths of CapsuleNet to achieve efficient lesion classification in gastric capsule endoscopy images. The selection of ResNet50 as the feature backbone is predicated on its robust capability in extracting local textural features while maintaining computational efficiency compared to deeper variants [18,20]. Simultaneously, the CapsuleNet branch models spatial hierarchies and pose information through dynamic routing [21]. This architecture allows for the capture of both two-dimensional textural features and spatial relationship data, addressing variations inherent in gastric capsule endoscopy like gastrointestinal motility. This hybrid architecture enables the integration of two-dimensional semantic features with spatial relationship data, addressing variations inherent in gastric capsule endoscopy such as gastrointestinal motility and patient posture. By optimizing the arrangement and quantity of these integrated components, DuDeM ensures accurate lesion detection under complex clinical conditions.

**Figure 1:**
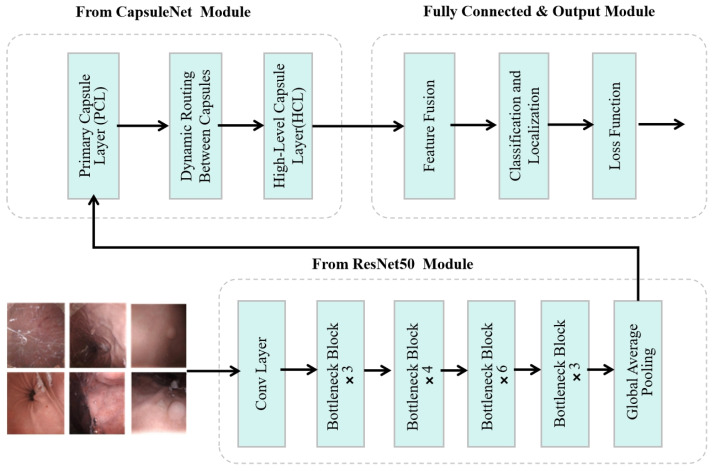
Architecture of the DuDeM model.

As shown in Figure 1, the diagnostic workflow of DuDeM follows a structured three-stage process: First, the input image is processed by the ResNet50-derived module, the core feature extractor. Comprising multiple convolutional layers and residual blocks, this stage captures mid-to-high-level semantic information—such as texture evolution and boundary discontinuities—providing the global perception necessary for robust lesion classification. Next, these features enter the CapsuleNet module, where capsule layers utilize vectorized representations to model spatial hierarchies and relative poses. This enhances robustness against postural perturbations and local occlusions via a dynamic routing module, which adaptively adjusts connection strengths based on the “prediction consistency” principle. This iterative mechanism enables structural reasoning and recognizes complex spatial combinations, mitigating information propagation errors common in fixed-weight networks. Finally, a weighted average fusion strategy integrates these independent semantic and spatial pathways into a unified feature stream. The output layer then executes two concurrent tasks: lesion classification, optimized via cross-entropy loss, and bounding box regression (center, width, and height), optimized via Smooth L1 loss. This end-to-end mechanism ensures the synergistic optimization of both diagnostic detection and localization accuracy.

#### 2.1.1. Overall Architecture of DuDeM

To address these limitations, DuDeM was designed with a CNN branch to capture local textures and contrast priors, and a Capsule branch to explicitly model pose and part-whole relationships. The outputs of these branches are fused at multiple scales using attention weights. The key steps of model integration include three stages: high-level feature extraction, spatial structure modeling, and feature fusion with classification. Given a standardized gastric capsule endoscopy image $x\in R^{480\times 480\times 3}$, the model predicts its class y ∈{1,…,K} (e.g., clinical examples: normal, erosion/ulcer/bleeding, polyp/protrusion). Denote the model’s predicted posterior class probabilities as $\hat{p}=f_{\theta }\left(x\right)\in \Delta^{K\;-1}$, where Δ denotes the learnable parameters and probability simplex and θ represents the learnable parameters. The objective is to minimize the expected risk:

(1) $$\underset{\theta }{min}E_{(x,y)∼D}\;L(f_{\theta }(x),y),$$

where D denotes the heterogeneous distribution of images collected from multiple hospitals, devices, and anatomical sites. The three stages of DuDeM are detailed as follows:

(1) ResNet50 Feature Extraction Stage

The DuDeM model incorporates the initial convolutional layer, max-pooling layer, four residual blocks, and a global average pooling layer of ResNet50. These components are employed to extract and filter local features of medical images, including lesion texture patterns, color, and edge variations. Structurally, the input image is processed sequentially through the initial convolution layer, max-pooling layer, and four residual blocks, resulting in a 2048-dimensional feature tensor. This tensor is subsequently processed through the global average pooling layer to generate a fixed-length feature vector, denoted as $F_{ResNet}\in R^{d}$, where $F_{ResNet}$ serves as the features extracted by the ResNet50 model, all of which belong to the vector space $R^{d}$ with dimension d, thereby laying the groundwork for subsequent CapsuleNet modeling and feature fusion.

The specific operations are as follows: First, the input image is processed by the initial convolutional layer of ResNet50, where feature extraction is performed using a 7 × 7 convolutional kernel with a stride of 2. Second, the resulting feature map undergoes a pooling operation with a 3 × 3 kernel employing max-pooling and a stride of 2. Third, the feature map enters the four residual blocks of ResNet50, each containing multiple convolutional operations to further extract features, ultimately yielding the feature tensor. The ReLU activation function and batch normalization are applied during processing.

(2) $$F_{Resnet}\in R^{C_{c}\times H_{c}\times {\;W}_{c}}.$$

(2) CapsuleNet Spatial Relationship Modeling Stage

In this stage, CapsuleNet receives either the input image or feature maps extracted from ResNet50. The model partitions the input feature map into local regions using a principal component localization mechanism, generating a primary capsule vector for each receptive field. Each primary capsule encodes two types of information: the presence of specific visual features within the local region, represented by the vector magnitude (larger magnitudes indicate higher confidence), and the spatial pose of the feature, represented by the vector direction, including position, scale, and rotation.

Local features captured by primary capsules are further abstracted into higher-level semantic features through dynamic routing. Higher-level capsules selectively receive outputs from primary capsules, which predict the potential states of higher-level capsules based on their pose information. When the predicted vectors align with higher-level capsule vectors, connection weights are strengthened. Through iterative computation, weights are adaptively adjusted according to the degree of match between predictions and the actual state of the higher-level capsule, and the weighted primary capsule vectors are aggregated to form the output of higher-level capsules.

This mechanism allows higher-level capsules to adjust vector directions in response to changes in object viewpoint, rotation, or translation, while maintaining stable vector magnitudes that represent feature presence. Consequently, CapsuleNet demonstrates superior robustness to rotations and pose variations compared with conventional CNNs [22,23]. The process emulates human visual cognition by integrating local features into global object representations, enabling higher-level capsules to encode more abstract target features and their overall pose. After dynamic routing, the output of higher-level capsules is mapped to a fixed-length feature vector, $F_{\mathrm{CapsuleNet}}\in R^{d}$, consolidating all CapsuleNet-extracted features into a d-dimensional vector space, where each capsule encodes the spatial and pose attributes of the object across d real-valued components. The input x is processed through convolution to generate the PrimaryCaps layer, yielding *N* capsules, each represented as a d-dimensional vector:

(3) $$U\;=\;\{u_{i}\in R^{d}{\}}_{i\;=\;1}^{N},u_{i}=\;\mathrm{squash}(z_{i})$$

where the squash function is defined as $\mathrm{squash}\left(z\right)=\frac{∥z{∥}^{2}}{1+∥z{∥}^{2}}\frac{z}{∥z∥}$, which compresses the vector length to the interval (0,1). The vector length represents the probability of the entity’s presence, and the vector orientation encodes its pose information. Each lower-level capsule makes predictions for category capsules via an affine transformation:

(4) $${\hat{u}}_{j|i}=W_{ij}u_{i},j\;=\;1,\ldots ,K.$$

The coupling coefficients $\left\{c_{ij}\right\}$ are updated through rounds of routing-by-agreement. The iterative routing rules are:

(5) $$s_{j}=\;\sum_{i}c_{ij}{\hat{u}}_{j|i},v_{j}=\;\mathrm{squash}(s_{j}),b_{ij}\leftarrow b_{ij}+{\hat{u}}_{j|i}\cdot v_{j},c_{ij}={\mathrm{softmax}}_{j}(b_{ij}),$$

yielding the set of category capsules $V=\{v_{j}\in {R^{d}}^{\prime }{\}}_{j\;=1}^{K}$, and then multi-scale upsampling and alignment operations are applied to form the pose-aware feature map $F_{\mathrm{CapsuleNet}}\in R^{C_{v}\times H_{c\;}\times W_{c}}$, which has the same spatial dimensions as $F_{Resnet}$.

(3) Feature Fusion and Classification Decision Stage

The feature fusion strategy employs a “weighted averaging” approach. After obtaining the two independent feature vectors $F_{ResNet}$ and $F_{\mathrm{CapsuleNet}}$, a fusion mechanism is designed to integrate the two types of information for classification and detection tasks. The fusion expression is:

(6) $$F_{fusion}=\;\alpha \cdot F_{ResNet}+(1\;-\;\alpha )\cdot F_{\mathrm{CapsuleNet}}$$

where α ∈ [0, 1] is the fusion coefficient, used to adjust the relative importance of the two features. Here, $F_{ResNet}$ and $F_{\mathrm{CapsuleNet}}$ denote the features extracted by the ResNet50 and CapsuleNet models, respectively. To jointly leverage local texture and pose relationships, a channel–spatial joint attention mechanism is introduced:

(7) $$\alpha \;=\;\sigma (MLP(GAP([F_{c};F_{v}])))\;\in \;(0,1{)}^{C_{c}},$$

(8) $$F_{fuse}=\alpha ⊙F_{c}+(1-\alpha )⊙\phi (F_{v}),$$

where [⋅;⋅] denotes channel-wise concatenation, *GAP* denotes global average pooling, σ is the sigmoid function, and ϕ is a 1 × 1 convolution used for channel alignment. Finally, after passing through *GAP* and the classification head, the output is obtained as:

(9) $$\hat{p}\;=\mathrm{softmax}(W\;\mathrm{GAP}(F_{fuse})\;+\;b).$$

The weighted averaging method was adopted based on five considerations. First, it facilitates feature scale alignment. Weighted averaging directly aligns the feature dimensions extracted by ResNet50 and CapsuleNet within the feature space, avoiding complex feature remapping and improving computational efficiency and engineering scalability. Second, it balances robustness and flexibility, allowing the relative contributions of semantic image features and spatial pose features to be adjusted. For instance, in medical images with blurred textures but clear structures, reducing the weight of semantic features can enhance the perception of spatial pose features. Third, it supports end-to-end training and differentiability, enabling global parameter optimization via gradient descent without introducing additional parameters or dimensions, thereby mitigating overfitting and allowing seamless integration into the network. Fourth, its effectiveness has been empirically validated. Preliminary experiments comparing multiple fusion strategies—including feature concatenation followed by dimensionality reduction, attention-based fusion, and gating mechanisms—demonstrated that weighted averaging maintains a simpler model structure while achieving competitive performance, particularly under small-sample or noisy conditions. Fifth, it enhances lesion classification and detection. The fused feature vector is fed into the DuDeM fully connected layer, enabling lesion classification via the Softmax output and precise lesion localization through bounding box regression.

#### 2.1.2. DuDeM Loss Function and Uncertainty Calibration

In the DuDeM model, the output encompasses both lesion classification and localization information, and thus the training objective must optimize classification accuracy and localization precision simultaneously. Accordingly, DuDeM employs a composite loss function comprising a classification loss and a localization regression loss:

(10) $$L_{total}=L_{\mathrm{classification}}+\;\lambda \cdot L_{\mathrm{regression}}$$

where λ is a hyperparameter balancing the contribution of the two tasks, $L_{\mathrm{classification}}$ denotes the classification loss, and $L_{\mathrm{regression}}$ denotes the localization regression loss.

Although not explicitly represented as a network module, the loss function serves as the fundamental driver of network convergence. Without it, the model cannot generate effective learning feedback, and network design is often constructed to optimize gradients derived from the loss. Functionally, the loss is as essential to the model as activation functions or connection weights. The classification loss enhances lesion recognition accuracy and is formally defined as:

(11) $$L_{\mathrm{classification}}=\;-\sum_{i\;=\;1}^{N}y_{i}log({\hat{y}}_{i})$$

where $y_{i}\in \{0,1\}$ indicates the true label of the i-th sample (e.g., whether it belongs to lesion type A), and ${\hat{y}}_{i}\in [0,1]$ is the predicted probability output by the model for a certain category, ranging between 0 and 1. This loss function is the log-likelihood loss commonly used in multi-class classification tasks, which minimizes the divergence between the predicted distribution and the true distribution.

The classification loss is compatible with probabilistic outputs and is commonly paired with Softmax or Sigmoid activations in the final layer. These combinations ensure stable gradient propagation and robust adaptation to class imbalance, which is prevalent in medical imaging datasets. Assigning higher weights to minority classes improves model sensitivity to rare lesion types, directly constraining the model’s discriminatory capacity. Gradients from the classification loss guide the network to adjust feature extraction, enhancing regions relevant to underrepresented lesion classes. The localization regression loss optimizes lesion positioning. It constrains the model to learn spatially precise features, particularly enhancing CapsuleNet’s sensitivity to object pose and structural edges. This loss is defined as:

(12) $$L_{\mathrm{regression}}=\sum_{i\;=1}^{N\;}{\mathrm{smooth}}_{L_{1}}\left(p_{i}-{\;t}_{i}\right)$$

where $p_{i}$ denotes the predicted bounding-box parameter (e.g., center coordinates, width, height) and $t_{i}$ is the corresponding ground-truth parameter. The regression loss quantifies the positional deviation between the predicted and ground-truth bounding boxes. This loss adopts the Smooth L_1_ loss, also known as the Huber loss function, defined as follows:

(13) $${smooth}_{L_{1}}\left(x\right)=\left\{\begin{array}{ll} 0.5x^{2}, & \mathrm{if}\;\left|x\right|<1\\ \left|x\right|-0.5, & \mathrm{otherwise} \end{array}\right.$$

The positional regression loss function provides both stability and robustness. Compared with the conventional L_2_ loss (which is sensitive to outliers) and the L_1_ loss (whose gradient is discontinuous), the Smooth L_1_ loss uses a quadratic term in regions of small error to ensure smoothness and a linear term in regions of large error to mitigate the risk of gradient explosion. Additionally, it offers advantages in adapting to small-sample settings and reducing localization ambiguity. In medical imaging, lesion regions are often small and have blurry boundaries, leading to considerable uncertainty in positional prediction. Employing a smooth regression loss facilitates stable convergence during model training and yields more reliable results. This loss directly optimizes the bounding-box parameters, enabling the model not only to recognize lesions but also to localize them precisely, thereby meeting the clinical demand for accurate spatial detection.

### 2.2. Experiments

In this section, the dataset used to train the DuDeM model is first briefly introduced, including the detailed steps involved in its construction, such as the sample size estimation method applied during patient data collection and the specific data acquisition procedures. Next, the DuDeM model is established, and its core components are presented. Finally, the training and validation processes of the model are described in detail.

#### 2.2.1. Sample Size Estimation

Sample size estimation was conducted in accordance with the requirements of the Guidelines for Good Clinical Practice of Medical Devices [24] and the Principles for Designing Clinical Trials of Medical Devices issued by the National Medical Products Administration [25]. To ensure rigor, two methods recommended in the guidelines were employed: the “single-arm target value trial sample size estimation” and the “diagnostic test sample size estimation.” Both methods estimate sample size based on the expected target accuracy, sensitivity, and specificity. The larger sample size calculated from the two methods was selected as the minimum required number of patients.

The single-arm target value trial sample size estimation is commonly used in trials where a parallel control group is unnecessary or infeasible. Its main parameter is the event rate, typically represented by clinically meaningful reference values such as accuracy. In this study, the clinically observed accuracy of physicians was used as the reference target value. The expected performance of the model was compared with the minimum acceptable target value, which was determined collaboratively by the researchers and clinical physicians during the study design phase. The sample size calculation formula is as follows:

(14) $$n=\frac{{\left[Z_{1-\;\alpha /2}\sqrt{P_{0}\left(1\;-\;P_{0}\right)}+{\;Z}_{1\;-\beta }\sqrt{P_{T}(1\;-{\;P}_{T})}\right]}^{2}}{(P_{T}\;-\;P_{0})2}$$

where *P_T_* is the expected accuracy of AI-based lesion detection, *P*_0_ is the minimum acceptable accuracy threshold, and *Z*_1−*α*/2_, *Z*_1−*β*_ are the standard normal distribution quantiles. When *α* = 0.05, *Z*_1−*α*/2_ = 1.96; when *Z*_1−*α*/2_ = 1.96, *Z*_1−*β*_ = 0.842. Based on evidence from prior studies and general clinical consensus [26,27,28], the minimum acceptable accuracy for lesion detection by physicians was set at 85% (*P*_0_ = 85%), and the expected accuracy of the trained ResNet50 base model was set at 95% (*P_T_* = 95%). Substituting these values yields *n* = 78. Considering a 20% dropout rate, the adjusted sample size was calculated as 78/0.8 = 97.5, rounded to 98 patients. Positive and negative cases were equally balanced (1:1), resulting in at least 98 positive and 98 negative patients, for a total of 196 participants.

The diagnostic test sample size estimation is typically used to validate the reliability of a novel diagnostic technique. Sensitivity is used to calculate the required sample size for the positive group, and specificity for the negative group. The calculation formula is:

(15) $$n=\frac{{P(1\;-\;P)(Z_{1\;-\;\alpha /2})}^{2}}{△}$$

In the above formula, *n* denotes the sample size of the positive group or the negative group, *Z*_1−*α*/2_ represents the quantile of the standard normal distribution, and *P* denotes the expected value of sensitivity or specificity. Based on interviews with clinical experts, the expected values of both sensitivity and specificity were set at 95%. △ represents the allowable margin of error for *P*, which is generally defined as one half of the width of the 95% confidence interval of *P*, with commonly used values ranging from 0.05 to 0.10. Substituting the above values into the formula yielded a required sample size of *n* = 73. Assuming a dropout rate of 20%, the adjusted sample size was rounded to 91. Therefore, at least 91 positive participants and 91 negative participants were required, yielding a total sample size of 182 participants. To ensure sufficient data and achieve or exceed the pre-specified targets, this study amplified the dataset by a factor of ten over the minimum required sample size. Consequently, at least 1960 gastric capsule endoscopy images were collected from patients to construct the dataset.

#### 2.2.2. Data Collection

##### Data Sources

This study constructed a disease-specific dataset by collaborating with tertiary public hospitals capable of performing MCCE. Historical imaging data were collected to build a high-quality EGC capsule endoscopy dataset. The collected data included gastric images, positional data, and image timestamps. This dataset was used to train and validate the DuDeM model in this study. Considerations for anatomical site selection of collected images: Capsule images were collected covering 10 gastric anatomical sites: gastric fundus, cardia, anterior and posterior walls of the gastric body, lesser curvature, greater curvature, gastric angle, anterior and posterior walls of the gastric angle, gastric antrum, lesser curvature of the antrum, and pylorus. Images from non-gastric sites were discarded.

Considerations for the quantity of images per anatomical site: Since the incidence of gastric lesions varies by location, the number of images collected per site also differed. The gastric antrum is a high-incidence site for gastric diseases, the pylorus connects the stomach and duodenum, and the cardia connects the stomach and esophagus, representing narrow passages. To clearly visualize lesions or abnormalities, more images were collected at these sites. The gastric fundus, having the largest area in contact with food and being susceptible to physical or chemical injury, also had a higher collection volume.

Hospitals needed to meet the following criteria to serve as data sources: (1) The hospital must have a standard MCCE-dedicated examination room and equipment. (2) The hospital must have a professional medical team experienced in MCCE procedures and have conducted related diagnostic and therapeutic services for at least five years. (3) The hospital must maintain a complete MCCE quality control (QC) system and have no MCCE-related medical incidents in the past three years. (4) The hospital must have a mature electronic medical record system and picture archiving and communication system, capable of complete data storage and efficient retrieval, with image formats conforming to industry standards. (5) Hospital selection must ensure geographic diversity to avoid regional data bias and ensure patient population diversity.

After preliminary evaluation and collaboration, this study selected nine tertiary public hospitals in the following provinces and municipalities in China: Beijing (Chinese PLA General Hospital; Beijing Chaoyang Hospital, Capital Medical University; Peking Union Medical College Hospital; and Tsinghua Changgung Hospital, Tsinghua University), Shanghai (First Affiliated Hospital of Naval Medical University and Ruijin Hospital), Shandong (Shandong Provincial Hospital), Guangdong (First Affiliated Hospital of Sun Yat-sen University), and Hubei (Tongji Hospital, Huazhong University of Science and Technology). The gastroenterology or digestive center departments were selected as collaboration units, with each completing at least 1000 MCCE cases annually. The data collection period was from 1 November 2021 to 31 December 2022.

##### Training and Test Set Division

**Figure 2:**
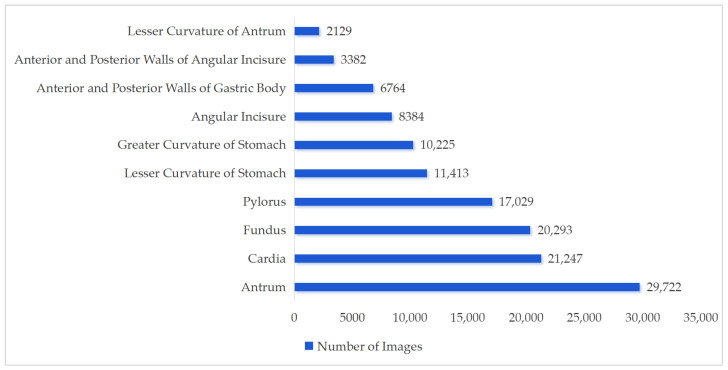
Number of images collected per gastric site.

**Figure 3:**
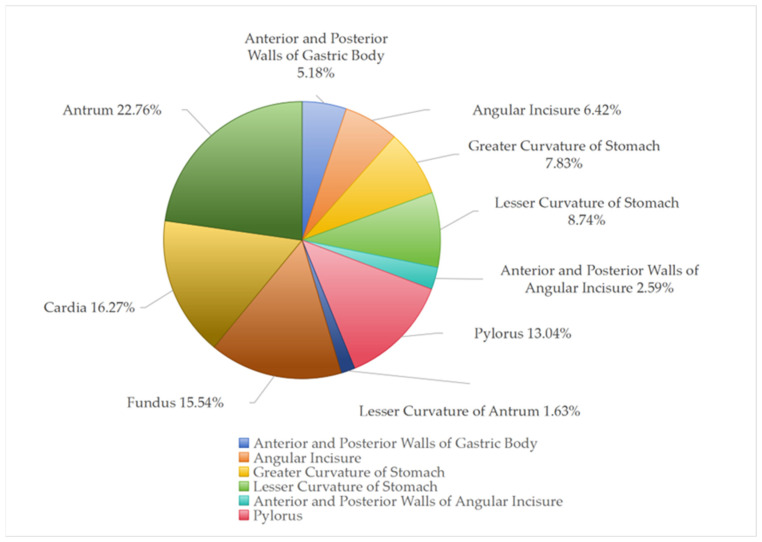
Proportion of images per gastric site.

A team of clinical experts annotated the collected images. A QC team then performed QC and arbitration on all annotated images. QC rules included: randomly selecting 20% of annotated images for re-examination; inconsistencies between the recheck and original annotation were resolved by QC personnel and arbitration experts. Images with ambiguous annotations were re-examined individually, and final decisions were made by QC personnel and arbitration experts. After annotation and QC, the final “EGC Capsule Endoscopy Dataset” consisted of 130,587 images from 2308 patients (exceeding the minimum required 1960 patients). Considering site-specific collection differences, the distribution of images by gastric site is shown in Figure 2 and Figure 3.

Figure 2 shows the number of images collected from each gastric site. The horizontal axis represents, in order: gastric fundus, cardia, anterior and posterior walls of gastric body, lesser curvature, greater curvature, gastric angle, anterior and posterior walls of gastric angle, gastric antrum, lesser curvature of antrum, pylorus. The vertical axis represents the number of images. The antrum had the highest number (29,722), while the lesser curvature of the antrum had the fewest (2129). The gastric antrum accounted for 22.76% of total images, followed by cardia 16.27%, fundus 15.54%, pylorus 13.04%, and other sites each under 10%.

**Figure 4:**
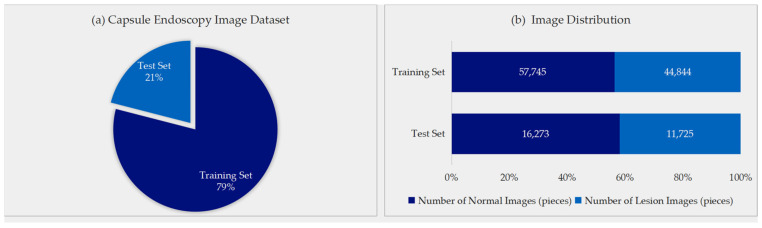
Distribution of images in the capsule endoscopy dataset.

**Figure 5:**
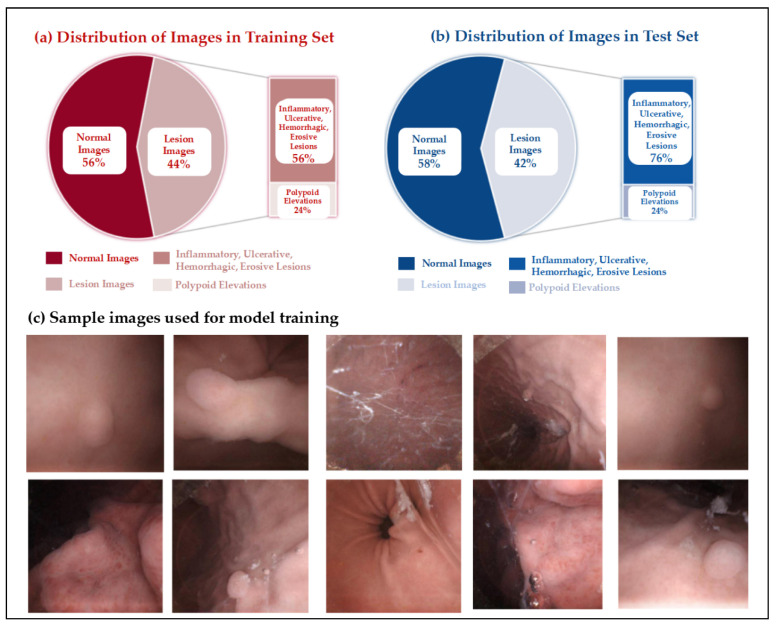
Distribution of non-tumor and polypoid lesion images in training and test sets. (**a**) Distribution of images in training set, (**b**) distribution of images in test set, (**c**) sample images used for model training.

Figure 4 shows the division of images per patient into training and test sets, which was ultimately divided as 79%:21% (approximately 4:1). The training set contained 102,589 images (57,745 normal, 44,844 lesion), and the test set contained 27,998 images (16,273 normal, 11,725 lesion). Figure 5a,b show the lesion image distribution in training and test sets. Normal images accounted for 56% in training sets and 58% in test sets, while lesion images accounted for 44% and 42%, respectively. Non-tumor lesions (inflammation, ulcer, bleeding, erosion) accounted for about 75%, and polypoid lesions (potential EGC) accounted for about 25%. The higher prevalence of benign non-tumor lesions explains this distribution. Examples of gastric site images collected, including normal anatomy (cardia, gastric body, antrum, pylorus) and lesion images (erosion, protrusions) were shown in Figure 5b. Image clarity, illumination, and contrast met requirements for model training and validation.

### 2.3. Model Development

#### 2.3.1. Model Training

The DuDeM model combines ResNet50 for detailed feature extraction and CapsuleNet for understanding spatial relationships between features. Its architecture includes six core components: input layer, base module, capsule layer, dynamic routing layer, feature fusion layer, and output layer. The functions and descriptions of these six core components are summarized in Table 1.

To evaluate DuDeM’s performance, eight additional classical image recognition neural networks were compared on the same public datasets (CIFAR-100, MNIST, GastroVision, Dataset-access-for-PLOS-ONE). The brief introduction of DuDeM and comparison models were as follows: (1) ResNet50: Baseline for DuDeM, deep CNN with residual blocks, strong local feature extraction. Widely used in medical image classification. (2) VGG16: Classic 16-layer CNN, simple but highly effective in medical image classification. (3) AlexNet: 8-layer CNN, validated the effectiveness of multilayer convolution for accuracy. (4) MobileNetV2: Uses depthwise separable convolution for lightweight feature extraction, reducing computation while maintaining accuracy. (5) YOLOv5: Efficient object detection model, fast for lesion localization and classification. (6) Faster R-CNN: Two-stage detection model with region proposal network (RPN), simplified for classification in this study. (7) CNN–Transformer: Hybrid model capturing local and long-range features. (8) MobileViT: Lightweight Vision Transformer variant optimized for edge devices, balancing performance and model size. (9) DuDeM: Combines ResNet50 for local features and CapsuleNet for spatial features, enhancing classification and lesion detection, potentially reducing misdiagnosis.

#### 2.3.2. Model Performance Evaluation Metrics

To comprehensively evaluate the performance and cost-effectiveness of different models, this study adopts commonly used evaluation metrics for deep learning tasks, including the following metrics: (1) Test accuracy, as the primary metric, to assess the overall classification performance of each model. (2) Additional performance metrics, including sensitivity, specificity, area under the ROC curve (AUC), and F1-score. (3) Average training loss, which quantifies the discrepancy between model predictions and ground-truth labels on the training dataset, reflecting the degree of model fitting. It is calculated by summing the loss values of all training samples and dividing by the total number of samples, representing the average error across the training set. (4) Model parameter count and inference speed, including the total number of parameters and single-image inference time for each model (including DuDeM), to evaluate computational cost. (5) Cost–performance indicators, including training time and inference time for each model used in comparison with DuDeM.

#### 2.3.3. Dataset Preprocessing

Standardized preprocessing and augmentation procedures were applied to each dataset to ensure input consistency and improve the robustness of the DuDeM model. The specific procedures for each dataset are outlined below: (1) CIFAR-100, containing 100 classes of color images, with an original resolution of 32 × 32 pixels, upsampled to 64 × 64 pixels before being input into the model. (2) MNIST, consisting of 10 classes of handwritten digits, with an original resolution of 28 × 28 pixels, upsampled to 64 × 64 pixels before model input. (3) GastroVision, a gastrointestinal endoscopy image dataset, with an original resolution of 480 × 480 pixels, downsampled to 224 × 224 pixels before input. (4) PLOS-ONE, a capsule endoscopy gastric lesion image dataset, with an original resolution of 480 × 480 pixels, downsampled to 224 × 224 pixels before input. For all datasets, random horizontal flipping, random cropping (padding = 4, i.e., padding 4 pixels on each side of the original image), and normalization were applied.

#### 2.3.4. Hyperparameter Settings

The following hyperparameters and optimization strategies were configured to ensure stable convergence during the training of the DuDeM model: (1) Training epochs: The model was trained for a fixed total of 100 epochs, with each epoch involving a complete pass over the training dataset. (2) Optimizer: The Adam optimizer was used for end-to-end training. Adam combines momentum-based first-order gradient estimation with adaptive second-order learning rate optimization, enabling stable convergence even under sparse gradient conditions. A momentum coefficient of 0.9 was adopted to accelerate convergence and reduce oscillation, with weight decay set to 1 × 10^−4^ for regularization. (3) Learning rate: The initial learning rate was set to 0.005. A cosine annealing strategy was employed to decay the learning rate to 1 × 10^−5^ over 100 epochs. During the early stage (epochs 0–50), the learning rate decreased gradually from 0.005 to approximately 0.0025 (cosine function from 0 to π/2). During the later stage (epochs 50–100), the learning rate decreased more rapidly to 1 × 10^−5^ (cosine function from π/2 to π). (4) Batch size: A batch size of 32 images was used for training. (5) Loss function: Cross-entropy loss was adopted to measure the discrepancy between the predicted probability distribution and the ground-truth label distribution. (6) Early stopping: Training was terminated early if no performance improvement was observed on the validation set for 10 consecutive epochs.

#### 2.3.5. Adaptation of Comparison Models

To ensure fair comparability across models, the components and parameters of the comparison models were adjusted by removing modules unrelated to feature extraction or classification and reducing parameter counts, thereby aligning their functional scope and scale with that of the DuDeM model: (1) ResNet50, VGG16, AlexNet, and MobileNetV2 were pretrained on the large-scale ImageNet dataset to learn general visual features such as edges, textures, colors, and shapes. These pretrained weights were used for initialization, and only the final classification head, including fully connected layers and activation functions, was replaced and fine-tuned for the target task. (2) YOLOv5, which consists of a backbone for feature extraction, a neck for multi-scale feature fusion, and a head for bounding box regression, class probability prediction, and confidence estimation, was modified by retaining only the backbone and head components while the neck module was removed. The classification layer was replaced to match the parameter scale of DuDeM, converting the framework into an image classification model that outputs class probabilities. (3) Faster R-CNN, originally a two-stage object detection model composed of a region proposal network (RPN) and a detection head that generates candidate regions and classifies them using pooled features, was simplified into a single-stage structure for multi-model comparison. This was achieved by removing regression branches within the RPN and retaining only feature extraction and classification components, with the classification layers fine-tuned to perform image-level classification only. (4) CNN–Transformer has been systematically evaluated on the GastroVision and Kvasir-Capsule datasets, achieving approximate performance values of 0.701 (precision), 0.724 (sensitivity), 0.690 (F1-score), and 0.724 (accuracy) on Kvasir-Capsule. Given that experimental settings, class definitions, and sample balancing can influence results, hyperparameters were re-tuned to reproduce results under the unified protocol of the four selected public datasets. (5) MobileViT_V2_050 is a lightweight MobileViT V2 variant incorporating quantization-aware training designed for resource-constrained environments such as mobile devices, providing a strong balance among F1-score, model size, and deployability with a size of only 1.70 MB. To ensure fair comparison, preprocessing and training protocols were aligned with other baselines, utilizing the native classification head for image-level classification and a multitask variant sharing a common backbone for lesion localization.

Each dataset was split into training and testing sets at an 8:2 ratio. The test set was used exclusively for final evaluation and not involved in hyperparameter tuning. From the training set, 10% of samples were randomly selected as a validation set for early stopping and overfitting prevention. Training was repeated three times using different random seeds, and results are reported as mean ± standard deviation.

#### 2.3.6. Experimental Environment for Model Comparison

The hardware and software environments used for model comparison were identical to those used for DuDeM training. The deep learning hardware configuration included six NVIDIA A100 GPUs for large-scale training, one NVIDIA GeForce RTX 4090 GPU for architecture tuning and inference speed testing, and an Intel Ultra 9 285 K CPU with at least 16 cores. Across this hardware platform, over 100 GPU-hours of training were accumulated, covering comprehensive comparative experiments of nine models (including DuDeM) on four public datasets.

#### 2.3.7. Ablation Study Design

**Figure 6:**
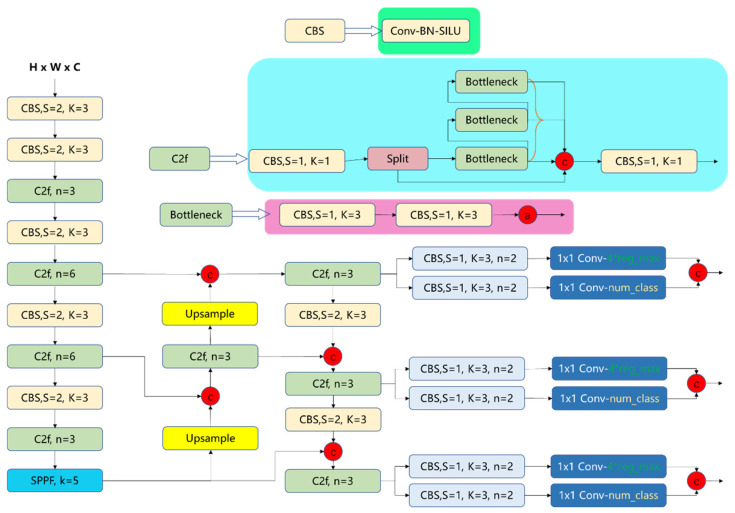
Ablation configuration of the DuDeM model. This figure illustrates how the DuDeM model validates the impact and necessity of different modules on model performance through ablation experiments. On the left is a backbone network for image feature extraction, which is used to extract multi-scale features from input images (with dimensions H × W × C). At the top is a feature enhancement module containing multiple Bottleneck structures, designed to enhance the extracted image features. At the bottom are multiple detection branches, each of which outputs classification results via a 1 × 1 convolutional layer to achieve lesion recognition. Yellow boxes represent Upsample modules; Green boxes denote C2f structures; Light blue boxes represent CBS blocks; and Blue boxes indicate SPPF and dark blue boxes represent detection heads. Red circles represent concatenation operations. The asterisk (*) denotes multiplication.

To quantify the contribution of each key component within the DuDeM model, a series of ablation experiments were conducted on four public datasets (MNIST, CIFAR-100, GastroVision, and PLOS-ONE). Core modules were removed or replaced individually, and performance changes were evaluated under identical training settings to clarify the role of each component across different visual tasks. To ensure feasibility after model decomposition, the DuDeM architecture was adjusted accordingly (Figure 6), with five validation objectives: (1) Evaluating the necessity of convolutional feature extractors in ResNet50. (2) Assessing the contribution of the dynamic routing mechanism in CapsuleNet to spatial relationship modeling. (3) Comparing feature fusion strategies, including attention-weighted fusion, element-wise summation, and direct concatenation. (4) Exploring the impact of pooling strategies, including max pooling and global average pooling, on classification performance. (5) Evaluating the effect of classification head depth, comparing single-layer and multi-layer fully connected designs.

The ablation configurations are summarized in Table 2.

Unified training and evaluation settings included: (1) Preprocessing and augmentation: Consistent with multi-model comparisons, with minor adjustments to image size, normalization, random flipping/rotation, and brightness/contrast perturbations. (2) Training protocol: Identical optimizer, learning rate schedule, batch size, number of epochs, and early stopping/regularization strategies; five fixed random seeds were used, reporting mean ± standard deviation. (3) Evaluation metrics: Accuracy, sensitivity, specificity, AUC, and F1-score; parameter count (M), floating-point operations, and single-GPU inference latency (ms/image) were also recorded. (4) Threshold selection: Determined by maximizing the Youden index on the validation set. (5) Statistical testing: Paired *t*-tests or Wilcoxon signed-rank tests were conducted comparing A0 with ablated variants and the baseline model (ResNet50). AUCs were evaluated using bootstrap 95% confidence intervals, with significance levels set at *p* < 0.05 and *p* < 0.01.

For each ablation group (A0–A6), models were independently trained and evaluated on all four datasets. To ensure result stability, each experiment was repeated three times, with mean ± standard deviation reported. Accuracy and loss curves were plotted uniformly to facilitate direct comparison between ablated models and the baseline.

## 3. Results

### 3.1. Multi-Model Comparison

**Figure 7:**
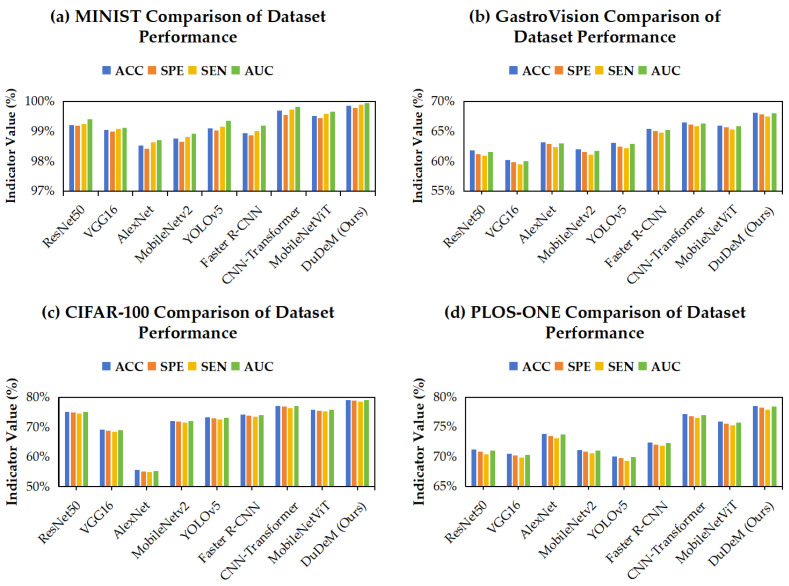
Performance comparison between the DuDeM model and mainstream CNN models across four datasets.

Table 3 presents the accuracy of different models across four datasets. On CIFAR-100, DuDeM achieved 78.82% test accuracy, outperforming ResNet50 (75.3%) by 3.5 percentage points and converging within 60 epochs. On MNIST, DuDeM reached 99.90%, slightly higher than MobileNetV2 (99.0%), with an inference time of 4.5 ms per image. On GastroVision and PLOS-ONE, DuDeM achieved 67.76% and 78.49%, respectively, exceeding other models by 4–8 percentage points, demonstrating robustness in medical imaging scenarios. Figure 7 shows the performance metrics (accuracy, specificity, sensitivity, and AUC) of DuDeM and eight mainstream CNN variants across the four datasets. DuDeM consistently achieved the highest scores in all metrics, highlighting its superior performance in both general and medical image scenarios.

DuDeM achieved the highest accuracy (ACC) across all four datasets, consistently outperforming classical CNN variants (ResNet50, VGG16, AlexNet, MobileNetV2). Compared with recent Transformer-based baselines (CNN–Transformer and MobileViT), DuDeM maintained superior performance, indicating that its fused strategy for local detail and pose feature extraction is effective in both general and medical imaging tasks. DuDeM’s specificity was consistently the highest or tied for highest across all experiments, on average about 3 percentage points higher than the next-best model. This indicates the lowest false-positive rate for negative samples (non-lesion/non-target regions), reducing misclassification of normal areas and potentially lowering clinical workload (fewer unnecessary follow-ups or manual reviews). Sensitivity was also consistently optimal, on average 4–5 percentage points higher than other models. High sensitivity reflects higher recall of positive samples (true lesions/targets) with fewer missed detections, including early-stage lesions and cases with positional or lighting variations, aligning with the clinical principle of “prefer over-reporting rather than under-reporting.”

**Figure 8:**
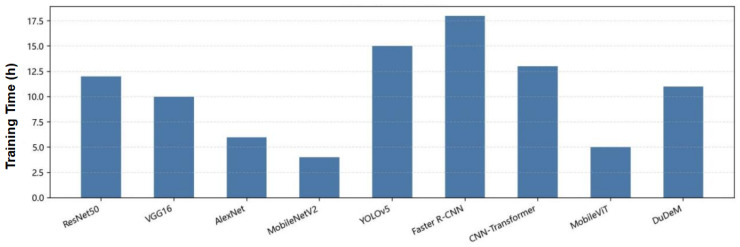
Comparison of training time across models.

**Figure 9:**
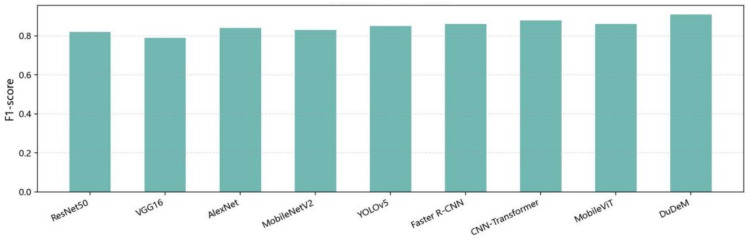
Comparison of F1-scores across models.

**Figure 10:**
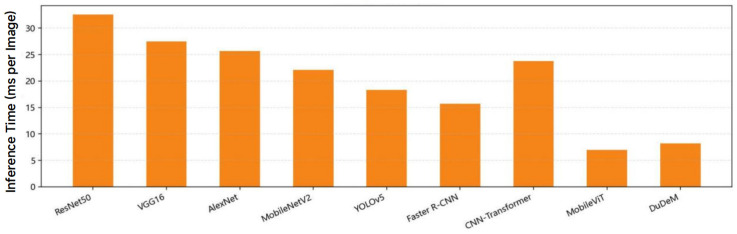
Comparison of inference time across models.

The mean AUC of DuDeM exceeded the next-best model by approximately 5 percentage points, with ROC curves closer to the top-left corner. This indicates stable and superior sensitivity-specificity trade-offs across different thresholds, ensuring good generalization and robustness over a broad operational range. Clinicians can thus flexibly adjust thresholds according to risk preferences without significantly compromising performance. Beyond performance metrics, DuDeM facilitates clinical interpretability by providing precise lesion localization through bounding box regression. This spatial evidence allows clinicians to verify the model’s diagnostic logic by identifying the specific ‘where’ behind each classification. Future iterations will explore the integration of Grad-CAM (Gradient-weighted Class Activation Mapping) to further visualize the specific attention regions driving the model’s decisions. The comparison of models on the general gastrointestinal dataset PLOS-ONE regarding F1-score, training time, and inference time is summarized in Table 4. Individual comparisons for training time, F1-score, and inference time across all evaluated models are respectively illustrated in Figure 8, Figure 9 and Figure 10.

The models’ F1-score ranking was as follows: DuDeM (0.91) > CNN–Transformer (0.88) ≈ MobileViT (0.86) ≈ Faster R-CNN (0.86) > YOLOv5 (0.85) > AlexNet (0.84) > MobileNetV2 (0.83) > ResNet50 (0.82) > VGG16 (0.79). DuDeM achieved the highest F1-score (0.91), consistently leading across all four datasets. ResNet50, as the baseline, scored 0.82, limited by missed and false detections of lesions. VGG16 (0.79), with shallower layers and weaker feature representation, had the lowest F1-score. AlexNet (0.84) slightly outperformed ResNet50 but lacked generalization in complex lesion regions. MobileNetV2 (0.83), designed for lightweight inference, traded off some classification and localization ability for faster mobile performance. YOLOv5 (0.85), benefiting from its object detection architecture, improved F1 slightly via enhanced localization. Faster R-CNN (0.86), with more precise region-level feature extraction, achieved the second-highest F1. Overall, DuDeM’s superior F1 confirms its strong performance.

DuDeM contains approximately 28 million parameters, comparable to ResNet50 (25 million). Despite a similar scale, DuDeM outperformed the baseline and most comparison models across accuracy, sensitivity, specificity, AUC, and F1, demonstrating higher “per-parameter efficiency” and a better trade-off among precision, efficiency, and stability. Its inference time per image was 8.2 ms, significantly faster than Faster R-CNN (15.7 ms), approaching real-time performance similar to MobileViT, while maintaining the highest F1. DuDeM simultaneously achieves very low false-positive rates (high specificity) and low false-negative rates (high sensitivity), making it well-suited for medical imaging applications. Its AUC results indicate robust classification performance across threshold adjustments, supporting flexible clinical operation.

The model’s dual-branch design, combined with attention-gated fusion, mitigates the limitations of conventional CNNs that focus only on local features and addresses pose invariance. The ResNet50 convolutional branch captures local texture and edge details, while the CapsuleNet branch, via routing-by-agreement, models global feature relationships and object pose, reducing misclassification due to posture, lighting, or gastric mucosa folds. The fusion layer aligns channel and spatial semantics, preventing conflicts between scalar features from CNN and vector features from CapsuleNet. Secondary training, regularization, and early stopping enhance generalization and robustness, reducing both false positives and false negatives.

DuDeM’s higher AUC across the four datasets demonstrates stable sensitivity–specificity balance under varying thresholds, enabling flexible operational points for clinical screening and diagnostic workflows. Combined with the highest F1 (0.91), near-fastest inference (8.2 ms per image), and parameter count comparable to ResNet50 (28 M), DuDeM shows deployable, scalable advantages in both general image recognition and gastric structure or lesion detection tasks, meeting clinical demands for high sensitivity, high specificity, and real-time performance.

### 3.2. Results of Model Ablation Experiments

**Figure 11:**
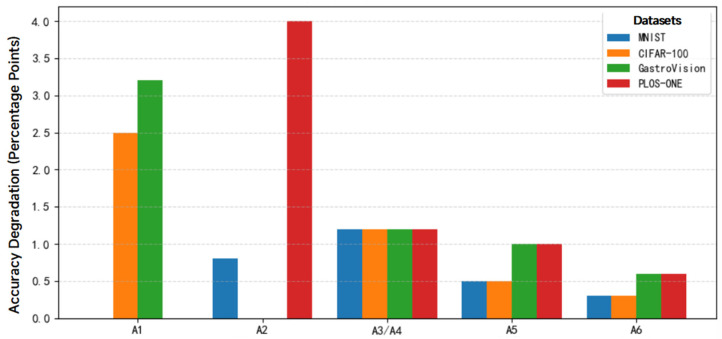
Performance degradation of DuDeM under different ablation settings.

Figure 11 compares the mean test accuracy and standard deviation of groups A0 and A1–A6 across four datasets. A0 represents the baseline DuDeM model and is treated as the reference point for calculating “performance degradation (percentage points).” Accordingly, the value for A0 is fixed at zero and corresponds to the horizontal line at y = 0 (the zero-degradation line). Only the relative performance drops of variants A1–A6 compared with A0 are plotted. This design enables quantitative assessment of the contribution of each module and identification of priority directions for optimization. In addition, the number of epochs required to reach a global test accuracy ≥95% was recorded, along with the average cross-entropy loss per epoch, to analyze model convergence speed and stability. As shown in Figure 11, accuracy drops of 2.5 and 3.2 percentage points were observed for the capsule module (A1 vs. A0) on CIFAR-100 and GastroVision, respectively, indicating that the dynamic routing mechanism plays a critical role in modeling spatial details. The ResNet module (A2 vs. A0) showed decreases of 0.8 and 4.0 percentage points on MNIST and PLOS-ONE, respectively, demonstrating the importance of convolutional feature extraction for complex image recognition tasks. For the feature fusion strategy (A3 vs. A4 vs. A0), A3 and A4 were on average 1.2 percentage points lower than A0, confirming that attention-weighted fusion outperforms simple summation or concatenation. The pooling strategy (A5 vs. A0) showed a decrease of 0.5–1.0 percentage points across all datasets, suggesting that global average pooling better integrates global information. The classification head depth (A6 vs. A0) exhibited a 0.3–0.6 percentage point decrease overall with slower convergence, indicating that deeper fully connected heads facilitate faster convergence. Through comparative training and evaluation of DuDeM against six mainstream CNN-based models and two state-of-the-art Transformer-based image processing models on four diverse datasets, DuDeM demonstrated faster convergence, higher classification accuracy, and lower inference latency. These results highlight the advantages and application potential of DuDeM in both general image recognition and medical image analysis.

Overall, DuDeM consistently achieved leading performance in terms of AUC, F1-score, sensitivity, and specificity across all four datasets. Performance gains were particularly pronounced on clinically relevant gastric image datasets such as GastroVision and PLOS-ONE. Even on the MNIST dataset, where performance approaches the theoretical upper bound, DuDeM maintained low variance outputs, reflecting strong convergence stability and reproducibility (mean ± standard deviation). Robustness evaluations under brightness, contrast, and random geometric perturbations showed performance fluctuations within acceptable ranges. Paired chi-square tests and Cohen’s kappa consistency analyses revealed no significant degradation, indicating favorable within-domain generalization. In this study, the robustness of DuDeM was evaluated under mild perturbations typical of clinical settings. Testing was not extended to extreme image degradation (e.g., severe motion blur or exposure failure), as such frames are clinically non-diagnostic and are pre-filtered during standard quality control. Prioritizing stability on diagnostic-grade images ensures the model’s reliability in practical screening workflows. Furthermore, through multi-branch feature fusion and parameter pruning, DuDeM achieved lower inference latency and resource consumption than most heavyweight detection or classification networks, supporting its potential deployment in both edge devices and clinical workstations. Collectively, DuDeM balances accuracy, efficiency, and robustness in general image recognition and medical imaging tasks such as magnetic-controlled capsule endoscopy, providing a solid foundation for future clinical decision support and large-scale screening systems.

### 3.3. Results of Secondary Generalization Experiments on Disease-Specific Datasets

In multi-model comparisons, DuDeM demonstrated superior recognition performance on both non-disease-specific general datasets and gastrointestinal datasets. To further enhance its capability for fine-grained structural recognition of EGC, a secondary fine-tuning strategy was adopted using the constructed EGC capsule endoscopy dataset. This secondary training aimed to improve recognition efficiency, accuracy, specificity, and sensitivity for disease-specific images, thereby laying the groundwork for clinical application in EGC screening.

**Figure 12:**
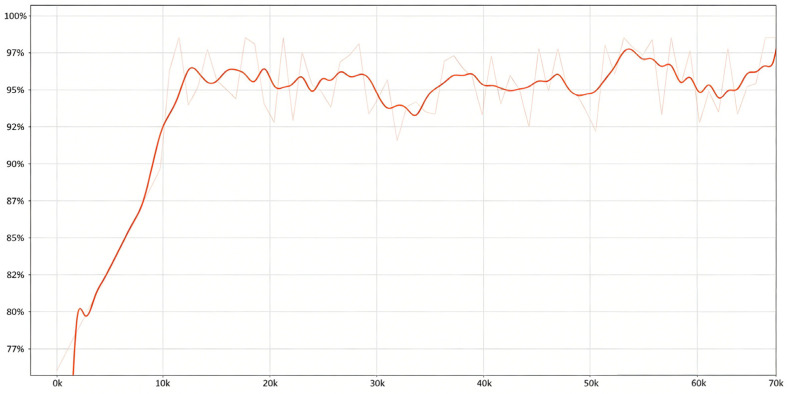
Accuracy curve of DuDeM during secondary training on the EGC capsule endoscopy dataset. The light red line indicates the raw accuracy per training batch, reflecting instantaneous fluctuations. The dark red line indicates the smoothed accuracy (moving average), illustrating the overall convergence and performance trend of the model.

Figure 12 illustrates the accuracy trajectory during secondary training, reflecting the dynamic evolution of model performance. The curve exhibits a “rapid increase followed by stabilization” pattern. During the early training phase (0 k–10 k iterations), accuracy rapidly increased to approximately 88%, indicating fast learning and effective fitting of the training data. In the mid-training phase (10 k–20 k iterations), accuracy further improved to approximately 98%. During the later phase (30 k–70 k iterations), accuracy fluctuated regularly around 96%, within the range of 93–98%, without further significant improvement. This suggests that model performance stabilized after approximately 30 k iterations, with additional training yielding no substantial gains while maintaining a high overall accuracy level.

**Figure 13:**
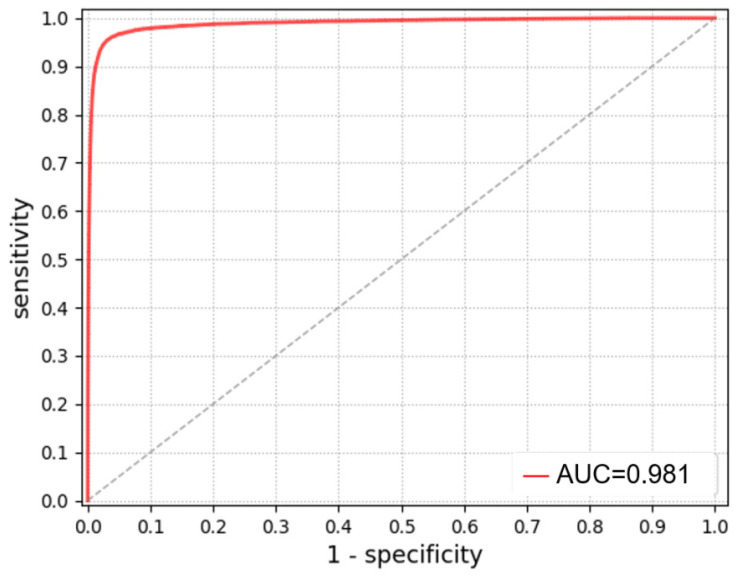
ROC curve of DuDeM after secondary training. The diagonal dashed line represents the performance of a random classifier (AUC = 0.5).

The ROC curve and AUC values after secondary training are shown in Figure 13. As the inflection point of the red ROC curve approaches the upper-left corner of the coordinate system, sensitivity (true positive rate) increases. The curve stabilizes near a sensitivity of 97.5%, while the false positive rate (1-specificity) corresponds to a specificity of 98.7%. The AUC, representing the area under the ROC curve and a key indicator of classification performance, reached 0.981, indicating excellent discriminative ability. The Youden index was calculated based on a sensitivity of 97.5% and specificity of 98.7%, yielding a value of 0.962, close to 1, indicating high classification validity and strong discrimination between lesion and non-lesion images. After secondary training, the model achieved a precision of 98.2% and an F1-score of 0.979.

As shown in Table 5, secondary training on the EGC capsule endoscopy dataset improved the AUC from 0.838 to 0.981 (+14.3 percentage points), indicating enhanced overall discriminative capability on disease-specific data. Sensitivity increased from 81.2% to 97.5%, reducing missed detections of EGC lesions. Specificity improved from 82.6% to 98.7%, reducing false positives for normal tissue. The Youden index increased from 0.638 to 0.962, reflecting improved balance between sensitivity and specificity. Precision rose from 77.1% to 98.2%, indicating a higher proportion of correctly identified lesions among positive predictions. The F1-score increased from 0.792 to 0.979, demonstrating substantial improvement in balancing false positives and false negatives. Analysis of the confusion matrix (Table 6) shows high diagonal values, indicating strong classification accuracy and consistency between predicted labels and ground-truth annotations.

**Figure 14:**
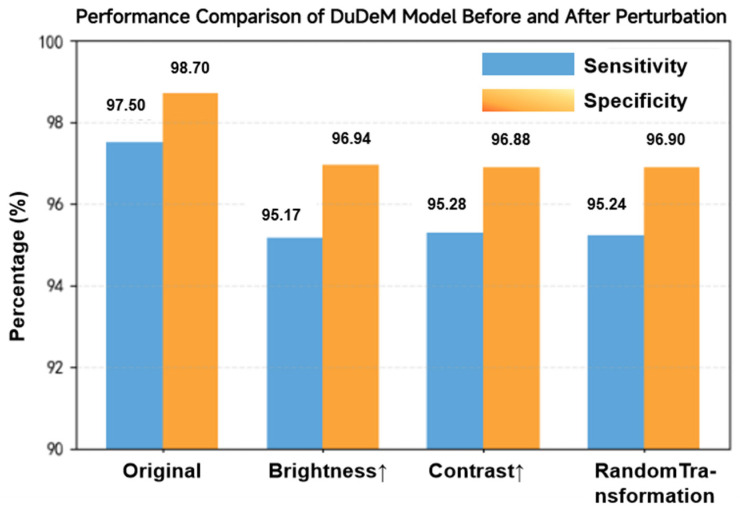
Performance comparison of DuDeM before and after perturbations. The arrows (↑) indicate an increase in image brightness and color contrast levels, respectively.

To further assess model stability, performance on the original test set was compared with three perturbation scenarios: changes in image brightness, changes in color contrast, and random basic transformations such as flipping (Figure 14). On the original test set, sensitivity and specificity were 97.50% and 98.70%, respectively. Under brightness perturbation, sensitivity decreased to 95.17% (−2.33 percentage points) and specificity to 96.94% (−1.76 percentage points). Under contrast perturbation, sensitivity decreased to 95.28% (−2.22 percentage points) and specificity to 96.88% (−1.82 percentage points). Under random transformations, sensitivity decreased to 95.24% (−2.26 percentage points) and specificity to 96.90% (−1.80 percentage points). Across all scenarios, including global brightness, contrast variations, and random geometric transformations, performance degradation remained below 3%, with sensitivity consistently above 95% and specificity showing even smaller declines (~1.8%). These findings indicate strong robustness of DuDeM to common image perturbations.

## 4. Conclusions

This study developed the DuDeM model for EGC detection in capsule endoscopy images, integrating complementary components from ResNet50 and CapsuleNet, with attention-weighted feature fusion to capture both local textures and pose relationships. A large-scale EGC capsule endoscopy dataset was constructed across nine tertiary hospitals, encompassing 2308 patients and ten gastric anatomical regions. While this repository addresses the scarcity of non-invasive endoscopy data, its primary reliance on a Chinese cohort necessitates further validation within international populations to ensure global generalizability. Additionally, as image acquisition was restricted to the Ankon MCCG system, future research will utilize diverse hardware platforms to confirm brand-agnostic compatibility and broad clinical applicability.

Under a unified training and evaluation framework, DuDeM was compared with eight mainstream models in terms of performance and efficiency, with additional ablation studies and robustness assessments under mild perturbations. Results demonstrated that DuDeM consistently outperformed comparative models in discriminative ability and stability. Secondary training on the disease-specific dataset further enhanced performance, with AUC increasing from 0.969 to 0.981, sensitivity from 96.1% to 97.5%, specificity from 97.3% to 98.7%, and the Youden index from 0.934 to 0.962. In summary, DuDeM achieves high accuracy, robustness, and interpretability without significant increases in parameter count or inference latency, providing a feasible solution for population-level EGC screening and supporting clinical decision-making.

## Figures and Tables

**Table 1:** Core components of DuDeM model.

Module	Function	Source Network	Description
Input Layer	Image preprocessing, low-level convolution	ResNet50	Uses Conv1, batch normalization, etc.
Base Module	Extract edges, textures, regions	ResNet50	Uses residual structure
Capsule Layer	Model pose, hierarchy, spatial relations	CapsuleNet	Vectorized representation
Dynamic Routing Layer	Adaptive update of capsule connections	CapsuleNet	Based on routing-by-agreement
Feature Fusion Layer	Feature combination and weighted averaging	Custom	Fuse outputs from both networks
Output Layer	Classification and position regression	Custom	Connects to loss function

**Table 2:** Design of ablation experiments.

Groups	Model Variant	Ablation/Modification	Objectives
A0	Full DuDeM	Original model	Performance baseline
A1	DuDeM–ResNet	Remove Capsule module	Assess Capsule contribution
A2	DuDeM–Capsule	Remove ResNet module	Assess necessity of ResNet features
A3	DuDeM–SumFusion	Replace attention fusion with summation	Compare fusion strategies
A4	DuDeM–ConcatFusion	Replace attention fusion with concatenation	Compare fusion strategies
A5	DuDeM–MaxPool	Replace all pooling with max pooling	Compare pooling strategies
A6	DuDeM–ShallowFC	Replace two-layer FC head with single-layer FC	Assess classification head depth

**Table 3:** Accuracy comparison (%).

Model	MNIST	CIFAR-100	GastroVision	PLOS-ONE
ResNet50	99.90 ± 0.10	75.30 ± 0.30	61.59 ± 0.27	71.09 ± 0.77
VGG16	99.65 ± 0.05	68.90 ± 0.40	60.28 ± 0.56	70.48 ± 0.52
AlexNet	99.04 ± 0.10	55.50 ± 0.50	63.10 ± 0.41	73.73 ± 0.81
MobileNetV2	99.00 ± 0.20	72.00 ± 0.30	61.89 ± 0.25	71.39 ± 0.43
YOLOv5	99.30 ± 0.10	73.10 ± 0.35	62.83 ± 0.09	70.10 ± 0.65
FasterR-CNN	99.10 ± 0.12	74.00 ± 0.30	65.16 ± 0.36	72.38 ± 0.63
CNN–Transformer	99.82 ± 0.06	76.95 ± 0.42	66.28 ± 0.39	76.92 ± 0.58
MobileViT	99.80 ± 0.06	75.80 ± 0.40	65.70 ± 0.34	75.90 ± 0.56
DuDeM	99.90± 0.05	78.82 ± 0.61	67.76 ± 0.45	78.49 ± 0.50

**Table 4:** Comparison of F1-score, training time, and inference time across models.

Model	F1-Score	Training Time (h)	Inference Time (ms/Image)
ResNet50	0.82	12	32.6
VGG16	0.79	10	27.5
AlexNet	0.84	6	25.7
MobileNetV2	0.83	4	22.1
YOLOv5	0.85	15	18.3
Faster R-CNN	0.86	18	15.7
CNN–Transformer	0.88	13	23.8
MobileViT	0.86	5	7
DuDeM	0.91	11	8.2

**Table 5:** Performance comparison of DuDeM before and after secondary training on the EGC dataset.

Metric	Pre-Training Generalization	Post-Training Generalization	Improvement
AUC	0.838	0.981	0.143
Sensitivity	81.20%	97.50%	16.30%
Specificity	82.60%	98.70%	16.10%
Youden index	0.638	0.962	0.324
Precision	77.10%	98.20%	21.10%
F1-score	0.792	0.979	0.187

**Table 6:** Confusion matrix after secondary training of DuDeM.

Ground Truth/Prediction	Normal	Inflammation/Ulcer/Bleeding/Erosion	Polyp/Protrusion
Normal	16,060	187	26
Inflammation/Ulcer/Bleeding/Erosion	151	8666	88
Polyp/Protrusion	144	194	2482

## Data Availability

The data used in this study are not publicly available due to patient privacy protection and institutional restrictions. De-identified data could be made available from the corresponding author upon reasonable request and subject to approval by the data-providing institutions.

## Abbreviations

The following abbreviations are used in this manuscript:

AI	Artificial Intelligence
EGC	Early Gastric Cancer
MCCE	Magnetically Controlled Capsule Endoscopy
CNN	Convolutional Neural Networks
DuDeM	DualNet Detection Model
AUC	Area Under the Receiver Operating Characteristic Curve
QC	Quality Control
